# Pilates for low back pain

**DOI:** 10.1590/1516-3180.20161344T1

**Published:** 2015-11-13

**Authors:** Tiê P. Yamato, Christopher G. Maher, Bruno T. Saragiotto, Mark J. Hancock, Raymond W. J. G. Ostelo, Cristina M. N. Cabral, Luciola C. Menezes Costa, Leonardo O. P. Costa

## Abstract

**BACKGROUND:**

Non-specific low back pain is a major health problem worldwide. Interventions based on exercises have been the most commonly used treatments for patients with this condition. Over the past few years, the Pilates method has been one of the most popular exercise programmes used in clinical practice.

**OBJECTIVES:**

To determine the effects of the Pilates method for patients with non-specific acute, subacute or chronic low back pain.

**METHODS:**

*Search methods:* We conducted the searches in CENTRAL, MEDLINE, EMBASE, CINAHL, PEDro and SPORTDiscus from the date of their inception to March 2014. We updated the search in June 2015 but these results have not yet been incorporated. We also searched the reference lists of eligible papers as well as six trial registry websites. We placed no limitations on language or date of publication.

*Selection criteria:* We only included randomized controlled trials that examined the effectiveness of Pilates intervention in adults with acute, subacute or chronic non-specific low back pain. The primary outcomes considered were pain, disability, global impression of recovery and quality of life.

*Data collection and analysis:* Two independent raters performed the assessment of risk of bias in the included studies using the 'Risk of bias' assessment tool recommended by The Cochrane Collaboration. We also assessed clinical relevance by scoring five questions related to this domain as 'yes', 'no' or 'unclear'. We evaluated the overall quality of evidence using the GRADE approach and for effect sizes we used three levels: small (mean difference (MD) < 10% of the scale), medium (MD 10% to 20% of the scale) or large (MD > 20% of the scale). We converted outcome measures to a common 0 to 100 scale when different scales were used

**MAIN RESULTS:**

The search retrieved 126 trials; 10 fulfilled the inclusion criteria and we included them in the review (a total sample of 510 participants). Seven studies were considered to have low risk of bias, and three were considered as high risk of bias.

A total of six trials compared Pilates to minimal intervention. There is low quality evidence that Pilates reduces pain compared with minimal intervention, with a medium effect size at short-term follow-up (less than three months after randomization) (MD -14.05, 95% confidence interval (CI) -18.91 to -9.19). For intermediate-term follow-up (at least three months but less than 12 months after randomization), two trials provided moderate quality evidence that Pilates reduces pain compared to minimal intervention, with a medium effect size (MD -10.54, 95% CI -18.46 to -2.62). Based on five trials, there is low quality evidence that Pilates improves disability compared with minimal intervention, with a small effect size at short-term follow-up (MD -7.95, 95% CI -13.23 to -2.67), and moderate quality evidence for an intermediate-term effect with a medium effect size (MD -11.17, 95% CI -18.41 to -3.92). Based on one trial and low quality evidence, a significant short-term effect with a small effect size was reported for function (MD 1.10, 95% CI 0.23 to 1.97) and global impression of recovery (MD 1.50, 95% CI 0.70 to 2.30), but not at intermediate-term follow-up for either outcome.

Four trials compared Pilates to other exercises. For the outcome pain, we presented the results as a narrative synthesis due to the high level of heterogeneity. At short-term follow-up, based on low quality evidence, two trials demonstrated a significant effect in favour of Pilates and one trial did not find a significant difference. At intermediate-term follow-up, based on low quality evidence, one trial reported a significant effect in favour of Pilates, and one trial reported a non-significant difference for this comparison. For disability, there is moderate quality evidence that there is no significant difference between Pilates and other exercise either in the short term (MD -3.29, 95% CI -6.82 to 0.24) or in the intermediate term (MD -0.91, 95% CI -5.02 to 3.20) based on two studies for each comparison. Based on low quality evidence and one trial, there was no significant difference in function between Pilates and other exercises at short-term follow-up (MD 0.10, 95% CI -2.44 to 2.64), but there was a significant effect in favour of other exercises for intermediate-term function, with a small effect size (MD -3.60, 95% CI -7.00 to -0.20). Global impression of recovery was not assessed in this comparison and none of the trials included quality of life outcomes. Two trials assessed adverse events in this review, one did not find any adverse events, and another reported minor events.

AUTHORS CONCLUSIONS: We did not find any high quality evidence for any of the treatment comparisons, outcomes or follow-up periods investigated. However, there is low to moderate quality evidence that Pilates is more effective than minimal intervention for pain and disability. When Pilates was compared with other exercises we found a small effect for function at intermediate-term follow-up. Thus, while there is some evidence for the effectiveness of Pilates for low back pain, there is no conclusive evidence that it is superior to other forms of exercises. The decision to use Pilates for low back pain may be based on the patient's or care provider's preferences, and costs.

The full text of this review is available from: http://onlinelibrary.wiley.com/doi/10.1002/14651858.CD010265.pub2/full
